# Improved crystallization and diffraction of caffeine-induced death suppressor protein 1 (Cid1)

**DOI:** 10.1107/S2053230X15001351

**Published:** 2015-02-21

**Authors:** Luke A. Yates, Benjamin P. Durrant, Michael Barber, Karl Harlos, Sophie Fleurdépine, Chris J. Norbury, Robert J. C. Gilbert

**Affiliations:** a Division of Structural Biology, Wellcome Trust Centre for Human Genetics, University of Oxford, Roosevelt Drive, Oxford OX3 7BN, England; b Sir William Dunn School of Pathology, University of Oxford, South Parks Road, Oxford OX1 3RE, England

**Keywords:** terminal uridylyltransferases, Cid1

## Abstract

The post-transcriptional addition of uridines to the 3′-end of RNAs is an important regulatory process that is critical for coding and noncoding RNA stability. In fission yeast and metazoans this untemplated 3′-uridylylation is catalysed by a single family of terminal uridylyltransferases (TUTs) whose members are adapted to specific RNA targets. In *Schizosaccharomyces pombe* the TUT Cid1 is responsible for the uridylylation of polyadenylated mRNAs, targeting them for destruction. In metazoans, the Cid1 orthologues ZCCHC6 and ZCCHC11 uridylate histone mRNAs, targeting them for degradation, but also uridylate microRNAs, altering their maturation. Cid1 has been studied as a model TUT that has provided insights into the larger and more complex metazoan enzyme system. In this paper, two strategies are described that led to improvements both in the crystallogenesis of Cid1 and in the resolution of diffraction by ∼1.5 Å. These advances have allowed high-resolution crystallo­graphic studies of this TUT system to be initiated.

## Abstract 2

The use of truncation and RNA-binding mutations of caffeine induced death suppressor protein 1 (Cid1) as a means to enhance crystallogenesis leading to an improvement of X-ray diffraction resolution by 1.5 Å is reported.

## 1. Introduction   

The addition of untemplated nucleotides to the 3′-ends of RNAs is a critical regulatory mechanism involved in a host of cellular and physiological processes including cell-cycle control, cell proliferation and differentiation, and embryonic development (Norbury, 2013 ▶; Yates *et al.*, 2013 ▶). The addition of uridylyl ribonucleotides to RNA has been shown to alter the stability of both coding mRNAs and noncoding RNAs (Norbury, 2013 ▶). Its role is critical for a number of regulatory pathways; for example, U6 snRNA 3′-end processing (Trippe *et al.*, 1998 ▶), cell cycle-dependent histone mRNA decay (Schmidt *et al.*, 2011 ▶), miRNA-directed RNA decay (Shen & Goodman, 2004 ▶) and (more recently identified) miRNA maturation (Hagan *et al.*, 2009 ▶; Heo *et al.*, 2009 ▶; Yates *et al.*, 2013 ▶) and mature miRNA silencing activity (Jones *et al.*, 2012 ▶).

The enzymes responsible for RNA uridylylation are the terminal RNA uridylyltransferases (TUTs), which belong to a conserved family of nucleotidyltransferases and are related to DNA polymerase β and poly(A) polymerase (Rissland *et al.*, 2007 ▶; Yates *et al.*, 2012 ▶). The *Schizosaccharomyces pombe* cytoplasmic TUT Cid1, discovered by its involvement in S–M checkpoint control, is an efficient and robust 3′-polyuridylation enzyme (Rissland *et al.*, 2007 ▶; Yates *et al.*, 2012 ▶) that regulates RNA decay through a novel exonucleolytic pathway of RNA metabolism (Rissland & Norbury, 2009 ▶). However, under certain biochemical conditions *in vitro* Cid1 is also a poly(A) polymerase (Rissland *et al.*, 2007 ▶). Cid1 is a 46 kDa cytoplasmic protein containing a nucleotidyltransferase motif, found in all members of the DNA Polβ superfamily, and a poly(A) polymerase (PAP)-associated motif. Several structures of Cid1 have been solved as an apo enzyme and bound to UTP and substrate mimics (Lunde *et al.*, 2012 ▶; Yates *et al.*, 2012 ▶; Munoz-Tello *et al.*, 2012 ▶, 2014 ▶). Cid1 is structurally related to the terminal uridylyltransferases from the protozoan *Trypanosoma brucei*, which are involved in RNA editing (Deng *et al.*, 2005 ▶; Stagno *et al.*, 2007 ▶, 2010 ▶; Aphasizhev *et al.*, 2004 ▶), and shares a common modality of UTP binding in the catalytic cleft. However, in Cid1 uracil recognition is mediated by His336 of the nucleotide-recognition motif (NRM; Lunde *et al.*, 2012 ▶; Munoz-Tello *et al.*, 2012 ▶; Yates *et al.*, 2012 ▶). This histidine in the NRM is absent in the trypanosomal TUTs but is invariant in the cytoplasmic TUT orthologues of Cid1 in mammals and other metazoans (Yates *et al.*, 2012 ▶). Furthermore, the equivalent His336 in higher eukaryotes is critical for UTP selectivity (Lapointe & Wickens, 2013 ▶).

In 2012, we reported two crystal structures of Cid1 solved and refined to 3.0 and 3.2 Å resolution in UTP-bound and apo states, respectively (Yates *et al.*, 2012 ▶). The crystals generated in our previous study were thin and radiation-sensitive and took >14 d to grow. In this paper, we present strategies to improve the crystallization and diffraction resolution of Cid1 for further high-resolution crystallographic studies.

## 2. Materials and methods   

### 2.1. Cloning and site-directed mutagenesis   

A pGEX6P-1 truncated Cid1 construct (tCid1) was generated as described in Rissland *et al.* (2007 ▶), and the K133A/R137A/R277A/K282A mutant was generated by successive site-directed mutagenesis procedures as described in Yates *et al.* (2012 ▶) using the primers described in Table 1 ▶, where the suffixes ‘F’ and ‘R’ signify forward and reverse primers, respectively, and underlined capitalized nucleotides denote mutagenized codons.

To generate the minimal Cid1 construct (mCid1), a DNA fragment encoding residues 41–377 was amplified using the pGEX6P-1-*tCid1* plasmid as a template using Phusion High-Fidelity DNA polymerase Master Mix (NEB) following the manufacturer’s instructions and using the primers in Table 1 ▶. The DNA fragment was purified and cloned into a pOPINF vector essentially as described by Bird (2011 ▶). The underlined sequence in the primers refers to the homologous region required for InFusion cloning. The amino-acid sequences of the proteins used in crystallization experiments are also given in conjunction with the primer sequences. All plasmids were sequence-verified prior to this study.

### 2.2. Expression of Cid1 in *Escherichia coli*   

*E. coli* BL21 (DE3) Star cells (Novagen) were transformed with either pGEX-6P-*tCid1* (and its mutants), which encodes a GST-tagged and N-terminally truncated (33–405) tCid1, or pOPINF-*mCid1*, which encodes an N-terminally His_6_-tagged minimal catalytic region (41–377). Transformants were selected for and cultivated on agar plates supplemented with 50 µg ml^−1^ carbenecillin and incubated at 310 K. Single colonies were picked, inoculated into LB supplemented with antibiotics and incubated at 310 K overnight with shaking at 200 rev min^−1^. Overnight cultures were diluted 1:100 into fresh Terrific Broth supplemented with antibiotics and auto-induction solution (Studier, 2005 ▶) and incubated at 310 K with shaking at 200 rev min^−1^ for ∼4 h. Protein expression was achieved by incubating cultures at 291–297 K for 12–18 h with shaking at 200 rev min^−1^. Bacteria were harvested by centrifugation at 5000*g* for 20 min at 277 K. The supernatant was decanted and the pellet was transferred and frozen at 253 K until use.

### 2.3. Purification of tCid1 and RNA-binding mutants   

Frozen bacterial cell pellets were thawed and resuspended in modified HEPES-buffered saline (HBS; 40 m*M* HEPES pH 7.0, 200 m*M* NaCl). The suspension was cooled in a salt–ice bath and the cells were disrupted *via* sonication (20 cycles of 5 s pulses and 10 s cooling periods; Sonics Vibra Cell sonicator) or *via* cell disruption at 25 kPa (Constant Systems cell disruptor). The lysate was clarified by centrifugation at 30 000–60 000*g* for 1 h at 277 K and the resulting supernatant was incubated with pre-equilibrated glutathione Sepharose 4B FF beads (GE Healthcare) at 277 K with gentle mixing overnight using the batch method. The beads, together with immobilized protein, were separated from the supernatant *via* a gravity-flow column (Bio-Rad), and the flowthrough was collected for SDS–PAGE analysis. The beads were washed with 20 column volumes of modified HBS and the wash flowthrough was collected for SDS–PAGE analysis. The washed glutathione Sepharose beads, with adsorbed protein, were incubated with PreScission protease (GE Healthcare) at 277 K for 24 h, thus liberating tCid1 from its fusion partner *in situ*. After 3C protease treatment, the cleaved product, tCid1, was collected in the eluate. For completeness, the beads were washed with a minimal volume of modified HBS so that the remaining tCid1 protein from the column volume could also be collected. The presence of tCid1 in the eluants, together with its purity, was assessed by SDS–PAGE.

All eluants containing tCid1 were assessed to be ∼90% pure by SDS–PAGE and were pooled and diluted in 40 m*M* HEPES pH 7.0 until the final NaCl concentration was equal to 50 m*M*, and were then concentrated to 1 ml using a centrifugal filter device with a 15 kDa molecular-weight cutoff. The concentrated tCid1 solution in 40 m*M* HEPES pH 7.0, 50 m*M* NaCl was loaded onto a pre-equilibrated 5 ml Heparin HP column (GE Healthcare) *via* an ÄKTA FPLC at a rate of 1 ml min^−1^ at room temperature. The column was washed with a low-NaCl buffer (40 m*M* HEPES pH 7.0, 50 m*M* NaCl), and the protein was eluted using a linear NaCl gradient from 0.05 to 2 *M*. After this step the eluted tCid1 was assessed to be >95% pure by SDS–PAGE.

Finally, purification of tCid1 to homogeneity was achieved by size-exclusion chromatography using either a Superdex 200 16/60 or a Superdex 75 16/60 column and 20 m*M* HEPES pH 7.0, 200 m*M* NaCl, 0.5 m*M* tris(2-carboxyethyl)phosphine (TCEP) at room temperature. Fractions containing tCid1 (>98% purity as assessed by SDS–PAGE) were pooled and concentrated to 13.5 mg ml^−1^ using a centrifugal filter device with a molecular-weight cutoff of 15 kDa (Millipore). Protein was used for crystallization or was flash-frozen in liquid nitrogen and stored at 193 K.

The RNA-binding mutant tCid1 was slightly less soluble than tCid1 and required the addition of glycerol to maintain solubility. Therefore, we purified the RNA-binding mutant analogously to tCid1 using buffers supplemented with 5%(*w*/*v*) glycerol.

### 2.4. Purification of mCid1   

Frozen bacterial pellets were thawed and prepared analogously to tCid1. The resulting clarified lysate supernatant was incubated with pre-equilibrated nickel-chelated Sepharose beads overnight at 277 K with gentle mixing. The beads and adsorbed protein were then collected *via* a gravity-flow column (Bio-Rad) and the beads were washed with 15 column volumes (CV) of modified HEPES-buffered saline (50 m*M* HEPES, 150 m*M* NaCl, 20 m*M* imidazole pH 7.0) before the protein was eluted using a stepwise gradient of imidazole (50, 100, 300, 500 and 800 m*M*). Each wash was collected and its protein composition was analysed by SDS–PAGE. Subsequently, eluted fractions containing mCid1 were pooled, concentrated and purified by heparin and size-exclusion chromatography analogously to tCid1. Purified mCid was judged to be >95% pure by SDS–PAGE, and the fractions were pooled and buffer-exchanged into crystallization buffer (12 m*M* HEPES, 150 m*M* NaCl, 1 m*M* TCEP pH 7.0) and concentrated to 9 mg ml^−1^. Protein was used for crystallization or was flash-frozen in liquid nitrogen and stored at 193 K.

### 2.5. Crystallization of Cid1   

Purified tCid1 RNA-binding mutant (K133A/R137A/R277A/K282A; Yates *et al.*, 2012 ▶) was concentrated in a centrifugal filter device with molecular-weight cutoff 10 kDa to 13.5 mg ml^−1^ prior to crystallization in 20 m*M* HEPES, 200 m*M* NaCl, 0.5 m*M* TCEP pH 7.0. Crystallization was carried out using nanolitre-scale sitting-drop vapour-diffusion methods in CrystalQuick 96-well plates (Greiner Bio-One) at a range of concentrations (7–13.5 mg ml^−1^). Crystals appeared and grew in both 25%(*w*/*v*) PEG 3350, 200 m*M* ammonium sulfate, 100 m*M* bis-tris pH 6.5 after 7–12 h and 100 m*M* sodium citrate tribasic dihydrate pH 5.5, 18%(*w*/*v*) PEG 3350 or 16%(*w*/*v*) PEG 8000 within 4 h, both at 298 K. Purified mCid1 was concentrated to 9 mg ml^−1^ in 12 m*M* HEPES, 150 m*M* NaCl, 1 m*M* TCEP pH 7.0 and crystallized in 10%(*w*/*v*) PEG 3350, 0.1 *M* HEPES pH 7.5, 0.2 *M* proline, in which crystals appeared after 4 d and grew to maximum size within 7 d.

### 2.6. X-ray data collection and analysis   

Single crystals were cryoprotected with 25%(*v*/*v*) glycerol or 25%(*w*/*v*) ethylene glycol and mother liquor prior to flash-cooling in liquid nitrogen. Several data sets were collected from each crystal on beamlines I24, I02 and I03 at Diamond Light Source (DLS), Didcot, England. Diffraction data were indexed, integrated and scaled using *XDS* (Kabsch, 2010 ▶) and *SCALA* (Evans, 2006 ▶) in the *xia*2 software package (Winter, 2010 ▶). A complete summary of statistics for the diffraction data is given in Table 2 ▶.

## 3. Results and discussion   

### 3.1. Purification of tCid1   

**Figure 1:**
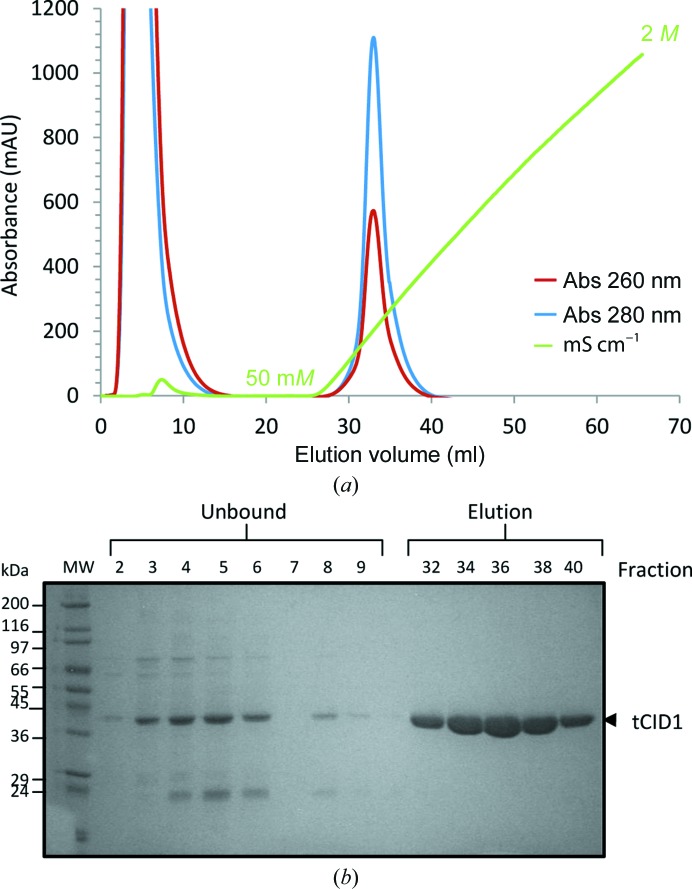
(*a*) Heparin affinity chromatography of tCid1 (without GST fused). The co-purified nucleic acids bound to the tCid1 enzyme (and mutants) were displaced by heparin affinity chromatography of tCid1. The protein that bound to the immobilized heparin was eluted using a linear sodium chloride gradient from 50 to 2 *M*. A single symmetrical peak eluted as a result of the sodium chloride gradient and clearly displays a significant absorbance at 280 nm and a greatly reduced 260:280 nm absorbance ratio. (*b*) SDS–PAGE analysis of the protein composition of the two peaks.

**Figure 2:**
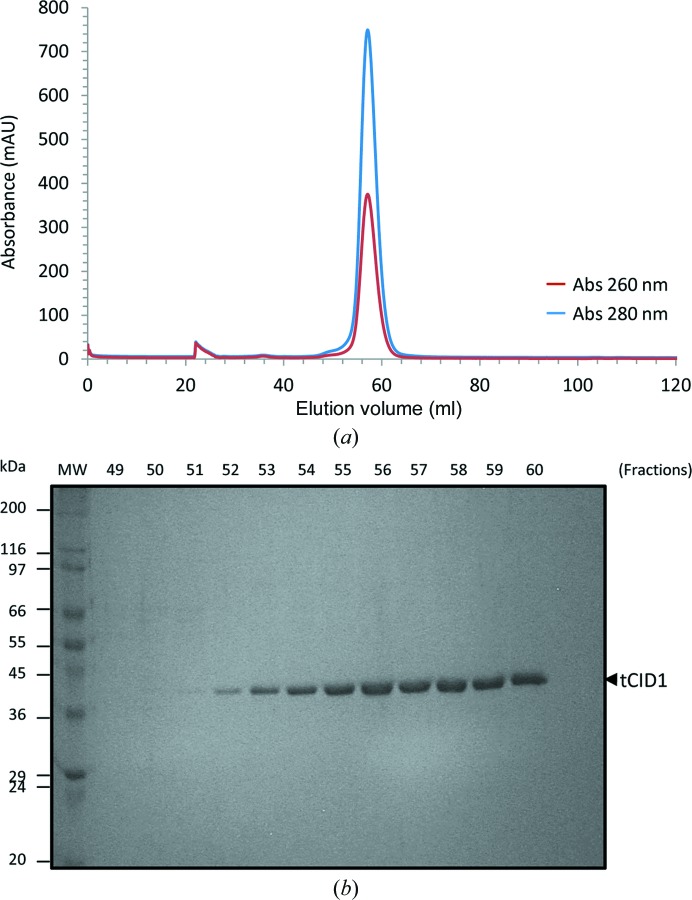
(*a*) Size-exclusion chromatography (SEC) of tCid1 (without GST fused or contaminating nucleic acids). A single symmetrical peak with a 260:280 nm ratio of 0.5 was observed when monitoring the elution at 280 and 260 nm. (*b*) SDS–PAGE analysis of the protein composition from SEC, demonstrating the presence of a single ∼45 kDa protein (tCid1).

We found that tCid1 co-purified with endogenous *E. coli* nucleic acids, which posed a significant problem for structural studies owing to induced sample heterogeneity and aggregation. We successfully displaced the bound nucleic acids using a heparin column as a pseudo-affinity step (Fig. 1 ▶). The resulting protein was then purified to homogeneity by size-exclusion chromatography (Fig. 2 ▶). We assessed the relative contamination of the protein with nucleic acids by calculating the absorbance ratio at wavelengths of 260 and 280 nm.

### 3.2. Previous crystallization of tCid1   

We screened ∼1000 crystallization conditions using commercially available sparse-matrix screens and 200 nl (100 nl protein plus 100 nl reservoir) sitting-drop vapour-diffusion experiments at two temperatures (298 and 277 K). Multiple plate-like crystals of tCid1 could be grown in Hampton Research Crystal Screen Cryo reagent No. 9 [15%(*v*/*v*) glycerol, 25.5%(*w*/*v*) PEG 4000, 170 m*M* ammonium acetate, 85 m*M* trisodium citrate pH 5.6] at room temperature, but they required optimization with volitaile organic compounds to generate crystals suitable for data collection. Even with our best optimization efforts these plate-like crystals diffracted to ∼3.2 Å resolution and were very radiation-sensitive, thus requiring microfocus beamlines to collect consecutive wedges of data to achieve a complete data set. Soaking with UTP improved the diffraction quality of these crystals, yielding 3.0 Å resolution data (Yates *et al.*, 2012 ▶). Therefore, we devised a number of strategies to improve both the success of crystallogenesis and the diffraction quality and resolution limit for further high-resolution studies.

### 3.3. Engineering a minimal Cid1 as a strategy to improve crystallization   

We engineered a minimal Cid1 (mCid1) that was truncated at both the N- and C-termini. The termini of this construct (residues 41–377) were based on the observable electron density of tCid1 (residues 33–405; PDB entry 4e7x; Yates *et al.*, 2012 ▶). We assumed that those residues that could not be reliably built at the termini were flexible and therefore were not necessary for crystallogenesis.

### 3.4. Surface mutation as a strategy to improve crystallization   

We made use of an RNA-binding mutant tCid1 construct that we had previously characterized (Yates *et al.*, 2012 ▶) for two reasons: (i) the mutated residues (K133A/R137A/R277A/K282A) could serve to the reduce the surface entropy of the protein, leading to improved crystallogenesis and alternative space groups, and (ii) to confirm that the lack of enzymatic activity observed in our earlier study (Yates *et al.*, 2012 ▶) was not caused by inducing protein unfolding or destabilization.

### 3.5. Crystallization of mCid1 and tCid1 RNA-binding mutant   

We again screened ∼960 crystallization conditions at two temperatures for each protein using nanolitre sitting-drop vapour-diffusion experiments as for tCid1 (see §[Sec sec3.2]3.2) and commercial sparse-matrix screens.

**Figure 3:**
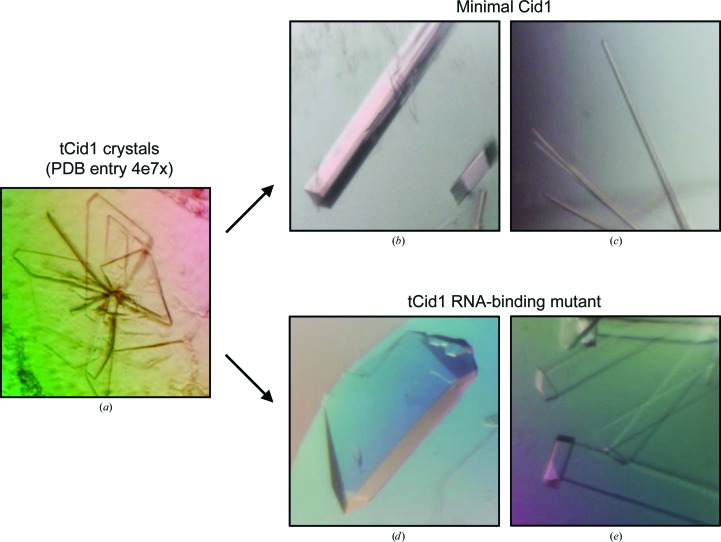
Crystals of (*a*) tCid1, produced to determine the structure with PDB code 4e7x (Yates *et al.*, 2012 ▶), (*b*) mCid1 crystal form I (space group *C*2), (*c*) mCid1 crystal form II (space group *P*2_1_), (*d*) tCid1 (K133A/R137A/R277A/K282A) mutant crystal form I (space group *P*1) and (*e*) tCid1 (K133A/R137A/R277A/K282A) mutant crystal form II (space group *P*2_1_). See Table 2 ▶ for further information.

Initial sparse-matrix screening of mCid1 yielded thin, needle-like crystals (550 µm in the longest direction) after 7 d in Hampton Research Index reagent No. 68 [10%(*w*/*v*) PEG 3350, 0.2 *M* HEPES pH 7.5, 0.2 *M* proline] at room temperature. We optimized the conditions by either (i) supplementing standard 200 nl sitting drops (100 nl protein plus 100 nl reservoir) with 50 nl of additives (Hampton Research, California, USA) or (ii) creating a dilution series of the original condition with distilled water in combination with increasing the drop size (to 300 nl) through alteration of the protein:reservoir ratio (1:1, 2:1 and 1:2 protein:reservoir). A total of around 240 drops were set up as optimizations and were kept at room temperature. Decreasing the concentration of PEG 3350 and maintaining the drop size decreased the time for crystals to appear from 7 to 4 d. Large single crystals (dimensions ∼215 × ∼15 × ∼15 µm) were grown alongside thin rod-like crystals by diluting the crystallization solution with distilled water to give the final condition 8.9%(*w*/*v*) PEG 3350, 178 m*M* HEPES pH 7.5, 178 m*M* proline (Figs. 3 ▶ *b* and 3 ▶ *c*).

We screened 960 crystallization conditions of the RNA-binding tCid1 mutant (K133A/R137A/R277A/K282A) at two temperatures using nanolitre sitting-drop vapour diffusion as for mCid1. Several conditions produced crystals which possessed two distinct morphologies. In the condition 25%(*w*/*v*) PEG 3350, 200 m*M* ammonium sulfate, 100 m*M* bis-tris pH 6.5 (Hampton Research Index reagent No. 67), large (dimensions ∼240 × ∼100 × ∼40 µm) single crystals appeared after 7 h and grew further within 12 h (Fig. 3 ▶ *e*). Crystals also grew in 100 m*M* sodium citrate tribasic dihydrate pH 5.5 with either 18%(*w*/*v*) PEG 3350 (Hampton Research PEGRx reagent No. 29) or 16%(*w*/*v*) PEG 8000 (Hampton Research PEGRx reagent No. 41) and appeared immediately after setting up the sitting-drop experiments before growing to completion within 4.5 h (largest dimensions ∼310 × ∼50 × ∼15 µm; Fig. 3 ▶ *e*). We optimized these conditions further by either supplementing the sitting drops with 50 nl of additives (Hampton Research) or by diluting the crystallization buffer with distilled water together with adjusting the drop ratio (1:1, 2:1 and 1:2 protein:reservoir). A total of around 240 drops were set up as optimizations and were kept at room temperature. However, the best diffracting crystals (see below) were grown from the original sparse-matrix condition.

We analysed the diffraction properties of the crystals using synchrotron X-ray radiation at Diamond Light Source, Didcot, England (see Table 2 ▶). We found that crystals grown using mCid1 diffracted to 2.25 Å resolution using synchrotron radiation, which was nearly a 1 Å improvement in resolution compared with those described previously (Yates *et al.*, 2012 ▶; see Table 2 ▶). Surprisingly, mCid1 crystallization produced two morphologically similar crystals that, on X-ray diffraction analysis, belonged to two different space groups. The larger crystals obtained belonged to space group *C*2, whereas some thin rods belonged to space group *P*2_1_ (see Table 2 ▶). The tCid1 (K133A/R137A/R277A/K282A) mutant crystallization experiments yielded two morphologically different crystal forms: large cube-like crystals and long plate-like crystals. X-ray diffraction showed that the two crystals belonged to two different space groups, *P*1 and *P*2_1_ (see Table 2 ▶). Satisfyingly, the largest cube-like crystals (space group *P*1) routinely diffracted to a resolution of better than 2.0 Å, with the highest resolution complete data set collected to 1.73 Å resolution, an improvement of ∼1.5 Å compared with ‘wild-type’ tCid1 crystals. The second crystal form (space group *P*2_1_), which possessed a plate-like morphology, diffracted to 2.51 Å resolution.

**Figure 4:**
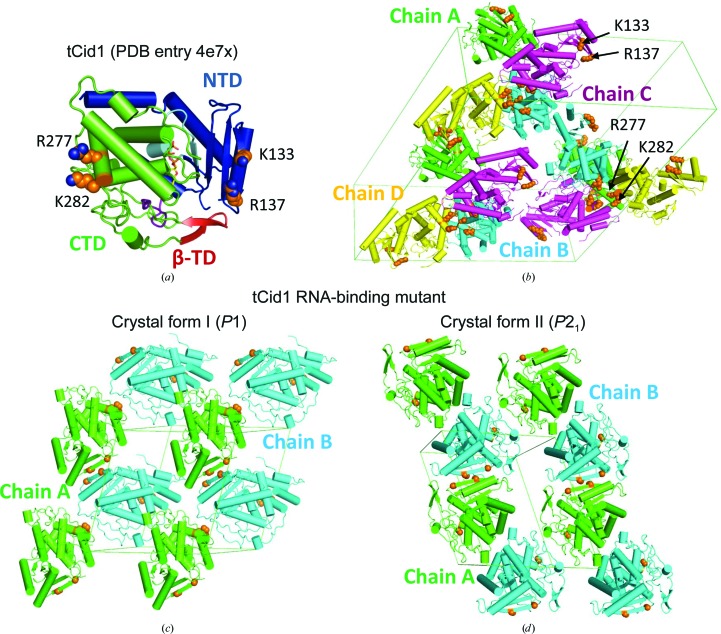
(*a*) Crystal structure of tCid1 (PDB entry 4e7x) highlighting the domain architecture, with the N-terminal domain (NTD) rendered in blue, the C-­terminal domain (CTD) rendered in green and the β-trap door (β-TD) feature of the structure which helps in substrate containment (Yates *et al.*, 2012 ▶, 2015 ▶) rendered in red. The sites that are mutated in our tCid1 RNA-binding mutant are shown as side chains (spheres) and are rendered in orange. (*b*) The crystal packing of PDB entry 4e7x showing the location of Lys133, Arg137, Arg277 and Lys282 and how this relates to crystal packing. This crystal structure possesses four molecules per asymmetric unit, which are rendered in green (chain *A*), cyan (chain *B*), purple (chain *C*) and yellow (chain *D*). In all chains the positions of Lys133, Arg137, Arg277 and Lys282 are rendered in orange and are shown as side chains (spheres). (*c*, *d*) The crystal packing of the RNA-binding mutant crystal structures in space groups *P*1 (*c*) and *P*2_1_ (*d*). The positions of K133A/R137A/R277A/K282A are highlighted in orange. Both crystal forms possess two molecules per asymmetric unit, which are rendered in green (chain *A*) and cyan (chain *B*) in both cases. This figure was generated using *PyMOL* (Schrödinger).

As we have already determined the crystal structure of tCid1 (PDB entry 4e7x; Yates *et al.*, 2012 ▶), we can visualize where the surface mutations sit (Fig. 4 ▶) and how this relates to the crystal packing in our first crystal form (space group *C*2; Fig. 4 ▶). Interestingly, the Arg277/Lys282 sites cluster together in PDB entry 4e7x, thus allowing four molecules in the asymmetric unit. As we can solve these RNA-binding mutant structures by molecular replacement, we can simply assess how the mutated sites relate to the crystal packing of these crystal forms. Clearly, the unit-cell parameters are smaller in these mutants compared with PDB entry 4e7x (see Table 2 ▶) and only two molecules are contained in the asymmetric unit. Indeed, calculating a Matthews coefficient for these crystal forms suggest that they all possess similar solvent contents (Table 2 ▶). Both R277A and K282A mutations favour packing against another molecule at a different site compared with the crystals yielding the structure with PDB code 4e7x (Fig. 4 ▶). However, we do still observe consistent packing interactions between the outer β-strands of the the N-terminal domains for PDB entry 4e7x and the tCid1 RNA-binding mutants in space groups *P*1 and *P*2_1_, despite these sites being mutated in the RNA-binding mutants (residues Lys133 and Arg137). In any case, the arrangement of molecules in the RNA-binding mutant crystal structure has given rise to another crystal form that can produce diffraction data to higher resolution (Table 2 ▶). It should be noted that the tCid1 RNA-binding mutant in space group *P*1 has undergone a large conformation change giving rise to this crystal form. The significance of this structure is described elsewhere (Yates *et al.*, 2015 ▶).

Using two different strategies, we have been able to improve the crystallization of Cid1 by either truncating the enzyme based on the observable electron density of the lower resolution structure or by using side-chain mutations on the surface of the enzyme, analogous to surface-entropy reduction (Derewenda & Vekilov, 2006 ▶). Furthermore, we have improved the resolution of diffraction by ∼1.5 Å compared with the original data, and this is helping us to pursue high-resolution studies of structure–function relationships in this enzyme. Similar strategies could be applied to other systems to achieve a more detailed understanding of enzyme function.

## Figures and Tables

**Table 1:** Summary of the constructs used in this study

tCid1 RNA-binding mutant	
Primer nucleotide sequences	
K133A/R137A-F	aggatttgaaggaGCatttttacaaGCggcaagaattccca
K133A/R137A-R	tgggaattcttgccGCttgtaaaaatGCtccttcaaatcct
R277A/K282A-F	cttcatggcttttttGCattttatgcttatGCgttcgagccacg
R277A/K282A-R	cgtggctcgaacGCataagcataaaatGCaaaaaagccatgaag
Amino-acid sequence of resulting protein†	GPLGSSYQKVPNSHKEFTKFCYEVYNEIKISDKEFKEKRAALDTLRLCLKRISPDAELVAFGSLESGLALKNSDMDLCVLMDSRVQSDTIALQFYEELIAEGFEGAFLQAARIPIIKLTSDTKNGFGASFQCDIGFNNRLAIHNTLLLSSYTKLDARLKPMVLLVKHWAKRKQINSPYFGTLSSYGYVLMVLYYLIHVIKPPVFPNLLLSPLKQEKIVDGFDVGFDDKLEDIPPSQNYSSLGSLLHGFFAFYAYAFEPREKVVTFRRPDGYLTKQEKGWTSATEHTGSADQIIKDRYILAIEDPFEISHNVGRTVSSSGLYRIRGEFMAASRLLNSRSYPIPYDSLFEEAPIPPRRQKKTDEQSNKKLLNETDGDNSE
Minimal Cid1 (mCid1)	
Primer nucleotide sequences	
Forward	aagttctgtttcagggcccgCACAAGGAATTTACGAAGTTTTGC
Reverse	atggtctagaaagctttaGGCCTCCTCAAATAATGAATCATAA
Amino-acid sequence of resulting protein†	MAHHHHHHSSGLEVLFQGPHKEFTKFCYEVYNEIKISDKEFKEKRAALDTLRLCLKRISPDAELVAFGSLESGLALKNSDMDLCVLMDSRVQSDTIALQFYEELIAEGFEGKFLQRARIPIIKLTSDTKNGFGASFQCDIGFNNRLAIHNTLLLSSYTKLDARLKPMVLLVKHWAKRKQINSPYFGTLSSYGYVLMVLYYLIHVIKPPVFPNLLLSPLKQEKIVDGFDVGFDDKLEDIPPSQNYSSLGSLLHGFFRFYAYKFEPREKVVTFRRPDGYLTKQEKGWTSATEHTGSADQIIKDRYILAIEDPFEISHNVGRTVSSSGLYRIRGEFMAASRLLNSRSYPIPYDSLFEEA

† Protein sequences are of the protein used for crystallization.

**Table 2:** Summary of data-collection statistics Values in parentheses are for the outermost resolution shell.

Protein	Tcid1 (PDB entry 4e7x)†	tCid1 (K133A/R137A/ R277A/K282A mutant) (crystal form I)	tCid1 (K133A/R137A/ R277A/K282A mutant) (crystal form II)	mCid1 (crystal form I)	mCid1 (crystal form II)
Crystallization condition	15%(*v*/*v*) glycerol, 25.5%(*w*/*v*) PEG 4000, 0.17*M* ammonium acetate, 0.085*M* trisodium citrate pH 5.6	25%(*w*/*v*) PEG 3350, 0.2*M* ammonium sulfate, 0.1*M* bis-tris pH 6.5	0.1*M* sodium citrate tribasic dihydrate pH 5.5, 18%(*w*/*v*) PEG 3350 or 16%(*w*/*v*) PEG 8000	10%(*w*/*v*) PEG 3350, 0.1*M* HEPES pH 7.5, 0.2*M* L-proline	10%(*w*/*v*) PEG 3350, 0.1*M* HEPES pH 7.5, 0.2*M* L-proline
Cryoprotectant‡	25%(*v*/*v*) glycerol	Stepwise increase to 25%(*v*/*v*) glycerol	25%(*v*/*v*) glycerol	Stepwise increase to 25%(*v*/*v*) glycerol	25%(*v*/*v*) ethylene glycol
Beamline	I24, DLS	I02, DLS	I02, DLS	I03, DLS	I03, DLS
Wavelength ()	0.973	0.9795	0.9795	0.963	0.963
Rotation per image ()	0.2	0.1	0.1	0.2	0.2
X-ray detector	PILATUS 6M	PILATUS 6M	PILATUS 6M	PILATUS 6M	PILATUS 6M
Temperature (K)	100	100	100	100	100
Space group	*C*2	*P*1	*P*2_1_	*C*2	*P*2_1_
*a*, *b*, *c* ()	164.4, 78.0, 152.5	58.96, 62.26, 65.5	62.7, 103.7, 76.3	157.1, 54.85, 119.6	116, 53.18, 124.6
, , ()	90, 109.5, 90	76.3, 81.1, 63.2	90, 110.8, 90	90, 125.6, 90	90, 115.2, 90
Resolution range ()	64.243.20 (3.223.20)	30.171.73 (1.771.73)	33.772.51 (2.582.51)	58.392.25 (2.312.25)	112.722.37 (2.432.37)
Molecules per ASU§	4	2	2	2	4
Solvent content¶ (%)	54	49	54	52	42
Total No. of reflections	130475	284535	104007	131393	190479
No. of unique reflections	30013	81044	30865	39354	56461
Completeness (%)	99.5 (97.7)	96.3 (95.2)	98.3 (98.8)	99.2 (99.5)	99.9 (99.9)
Multiplicity	4.1 (4.1)	3.5 (3.4)	3.4 (3.5)	3.3 (3.4)	3.4 (3.4)
*I*/(*I*)	8.9 (2.4)	16.7 (2.0)	22.6 (2.1)	15.3 (2.5)	8.3 (1.9)
*R*_merge_ ††	0.138 (0.712)	0.028 (0.528)	0.032 (0.531)	0.041 (0.494)	0.115 (0.630)

† Data for comparative purposes taken from Yates *et al.* (2012 ▶).

‡ Cryoprotectant was added to the reservoir solution of the crystallization experiment to the final concentration stated.

§ The number of molecules in the asymmetric unit (ASU) was confirmed by molecular replacement using *Phaser* (McCoy *et al.*, 2007 ▶) and PDB entry 4e7x.

¶ Solvent content calculated using the Matthews coefficient calculator (Weichenberger Rupp, 2014 ▶; Matthews, 1968 ▶). The molecular weight was derived from the sequences given in Table 1 ▶.

†† *R*_merge_ = 100 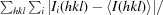 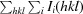, where *I_i_*(*hkl*) is the *i*th measurement and *I*(*hkl*) is the weighted mean of all measurements of *I*(*hkl*) for the reflection with Miller indices *hkl*.

## References

[bb1] Aphasizhev, R., Aphasizheva, I. & Simpson, L. (2004). *FEBS Lett.* **572**, 15–18. 10.1016/j.febslet.2004.07.004 15304317

[bb2] Bird, L. E. (2011). *Methods*, **55**, 29–37. 10.1016/j.ymeth.2011.08.002 21856427

[bb3] Deng, J., Ernst, N. L., Turley, S., Stuart, K. D. & Hol, W. G. J. (2005). *EMBO J.* **24**, 4007–4017. 10.1038/sj.emboj.7600861 PMC1356302 16281058

[bb4] Derewenda, Z. S. & Vekilov, P. G. (2006). *Acta Cryst.* D**62**, 116–124. 10.1107/S0907444905035237 16369101

[bb5] Evans, P. (2006). *Acta Cryst.* D**62**, 72–82. 10.1107/S0907444905036693 16369096

[bb6] Hagan, J. P., Piskounova, E. & Gregory, R. I. (2009). *Nature Struct. Mol. Biol.* **16**, 1021–1025. 10.1038/nsmb.1676 PMC2758923 19713958

[bb7] Heo, I., Joo, C., Kim, Y.-K., Ha, M., Yoon, M.-J., Cho, J., Yeom, K.-H., Han, J. & Kim, V. N. (2009). *Cell*, **138**, 696–708. 10.1016/j.cell.2009.08.002 19703396

[bb8] Jones, M. R., Blahna, M. T., Kozlowski, E., Matsuura, K. Y., Ferrari, J. D., Morris, S. A., Powers, J. T., Daley, G. Q., Quinton, L. J. & Mizgerd, J. P. (2012). *PLoS Genet.* **8**, e1003105. 10.1371/journal.pgen.1003105 PMC3510031 23209448

[bb9] Kabsch, W. (2010). *Acta Cryst.* D**66**, 125–132. 10.1107/S0907444909047337 PMC2815665 20124692

[bb10] Lapointe, C. P. & Wickens, M. (2013). *J. Biol. Chem.* **288**, 20723–20733. 10.1074/jbc.M113.455451 PMC3711335 23709223

[bb11] Lunde, B. M., Magler, I. & Meinhart, A. (2012). *Nucleic Acids Res.* **40**, 9815–9824. 10.1093/nar/gks740 PMC3479196 22885303

[bb12] Matthews, B. W. (1968). *J. Mol. Biol.* **33**, 491–497. 10.1016/0022-2836(68)90205-2 5700707

[bb13] McCoy, A. J., Grosse-Kunstleve, R. W., Adams, P. D., Winn, M. D., Storoni, L. C. & Read, R. J. (2007). *J. Appl. Cryst.* **40**, 658–674. 10.1107/S0021889807021206 PMC2483472 19461840

[bb14] Munoz-Tello, P., Gabus, C. & Thore, S. (2012). *Structure*, **20**, 977–986. 10.1016/j.str.2012.04.006 22608966

[bb15] Munoz-Tello, P., Gabus, C. & Thore, S. (2014). *Nucleic Acids Res.* **42**, 3372–3380. 10.1093/nar/gkt1278 PMC3950679 24322298

[bb16] Norbury, C. (2013). *Nature Rev. Mol. Cell Biol.* **14**, 643–653. 10.1038/nrm3645 23989958

[bb17] Rissland, O. S., Mikulasova, A. & Norbury, C. J. (2007). *Mol. Cell. Biol.* **27**, 3612–3624. 10.1128/MCB.02209-06 PMC1899984 17353264

[bb18] Rissland, O. S. & Norbury, C. J. (2009). *Nature Struct. Mol. Biol.* **16**, 616–623. 10.1038/nsmb.1601 PMC2875167 19430462

[bb19] Schmidt, M. J., West, S. & Norbury, C. J. (2011). *RNA*, **17**, 39–44. 10.1261/rna.2252511 PMC3004064 21051505

[bb20] Shen, B. & Goodman, H .M. (2004). *Science*, **306**, 997. 10.1126/science.1103521 15528436

[bb21] Stagno, J., Aphasizheva, I., Bruystens, J., Luecke, H. & Aphasizhev, R. (2010). *J. Mol. Biol.* **399**, 464–475. 10.1016/j.jmb.2010.04.021 PMC2916031 20403364

[bb22] Stagno, J., Aphasizheva, I., Rosengarth, A., Luecke, H. & Aphasizhev, R. (2007). *J. Mol. Biol.* **366**, 882–899. 10.1016/j.jmb.2006.11.065 PMC1850106 17189640

[bb23] Studier, F. W. (2005). *Protein Expr. Purif.* **41**, 207–234. 10.1016/j.pep.2005.01.016 15915565

[bb24] Trippe, R., Sandrock, B. & Benecke, B. J. (1998). *Nucleic Acids Res.* **26**, 3119–3126. 10.1093/nar/26.13.3119 PMC147682 9628908

[bb25] Weichenberger, C. X. & Rupp, B. (2014). *Acta Cryst.* D**70**, 1579–1588. 10.1107/S1399004714005550 24914969

[bb26] Winter, G. (2010). *J. Appl. Cryst.* **43**, 186–190.

[bb27] Yates, L. A., Durrant, B. P., Fleurdepine, S., Harlos, K., Norbury, C. J. & Gilbert, R. J. C. (2015). *Nucleic Acids Res.* 10.1093/nar/gkv122. 10.1093/nar/gkv122 PMC4357723 25712096

[bb28] Yates, L. A., Fleurdépine, S., Rissland, O. S., De Colibus, L., Harlos, K., Norbury, C. J. & Gilbert, R. J. (2012). *Nature Struct. Mol. Biol.* **19**, 782–787. 10.1038/nsmb.2329 PMC4342108 22751018

[bb29] Yates, L. A., Norbury, C. J. & Gilbert, R. J. (2013). *Cell*, **153**, 516–519. 10.1016/j.cell.2013.04.003 23622238

